# Acute Traumatic Spinal Cord Injury in Humans, Dogs, and Other Mammals: The Under-appreciated Role of the Dura

**DOI:** 10.3389/fneur.2021.629445

**Published:** 2021-02-03

**Authors:** Samira Saadoun, Nicolas D. Jeffery

**Affiliations:** ^1^Academic Neurosurgery Unit, St. George's, University of London, London, United Kingdom; ^2^Department of Small Animal Clinical Sciences, Texas A&M University, College Station, TX, United States

**Keywords:** animal, dog, dura, duroplasty, durotomy, human, spinal cord injury, surgery

## Abstract

We review human and animal studies to determine whether, after severe spinal cord injury (SCI), the cord swells against the inelastic dura. Evidence from rodent models suggests that the cord swells because of edema and intraparenchymal hemorrhage and because the pia becomes damaged and does not restrict cord expansion. Human cohort studies based on serial MRIs and measurements of elevated intraspinal pressure at the injury site also suggest that the swollen cord is compressed against dura. In dogs, SCI commonly results from intervertebral disc herniation with evidence that durotomy provides additional functional benefit to conventional (extradural) decompressive surgery. Investigations utilizing rodent and pig models of SCI report that the cord swells after injury and that durotomy is beneficial by reducing cord pressure, cord inflammation, and syrinx formation. A human MRI study concluded that, after extensive bony decompression, cord compression against the dura may only occur in a small number of patients. We conclude that the benefit of routinely opening the dura after SCI is only supported by animal and level III human studies. Two randomized, controlled trials, one in humans and one in dogs, are being set up to provide Level I evidence.

## Introduction

This review discusses acute traumatic spinal cord injury (SCI) with emphasis on cord swelling. We compare SCI in various mammals and present evidence for and against the notion that, after SCI, the cord swells against the dura. We then discuss two randomized, controlled trials (RCTs) being set up to define the role of the dura in human and dog SCI.

## Spinal Cord Injury in Mammals

### Humans

SCI is a devastating condition affecting about 180,000 patients annually worldwide (1). The commonest causes are road traffic accidents, sport injuries and falls. In younger patients, SCI is often associated with fracture or dislocation. Many older patients develop ‘central cord syndrome', a type of cervical incomplete SCI that occurs with pre-existing canal stenosis often in the absence of a fracture, causing primarily upper limb weakness (2). After SCI, most patients remain neurologically impaired with limb weakness or paralysis, loss of sensation below the injury, impaired urination, defecation and sexual function (3). To date, there is no treatment in clinical use proven to improve outcome after SCI.

### Dogs

SCI is commonly seen in dogs: veterinary clinics with appropriate imaging and surgical facilities treat >200 dog SCI cases per year and there are >200 such centers in the USA alone (4). The commonest cause of SCI is intervertebral disc prolapse, often occurring between T11–L3 vertebrae. Disc prolapse occurs in all dogs but is most common in chondrodystrophic individuals and so is especially prevalent in small breeds, notably the dachshund (5, 6). Susceptibility to disc degeneration is gene- and family-associated, with some dachshund families having >50% symptomatic individuals (7) and specific genetic determinants have recently been identified (8, 9). The severity of SCI varies, but about 20% of the total develop complete motor and sensory loss below the level of a thoracolumbar lesion (10). Furthermore, 10–15% of dogs with acute complete loss of motor and sensory function develop progressive myelomalacia, which appears almost unique in this species (11, 12). Progressive myelomalacia causes loss of neurological function that progresses caudally (to affect the hind limb reflex centers), cranially (to affect the intercostal muscle innervation or even the forelegs) or both. The cause is unknown, though imaging and *post-mortem* examination support progressive vascular injury and hemorrhage (13).

### Other Mammals

Our knowledge of SCI in other mammals is derived from their use in research. SCI is predominantly modeled in rodents, although some centers investigate primates, primarily because they provide better read-outs of hand function (14) and an increasing number utilize pigs (15). Most animal models (AMs) are contusive, dropping a weight or using an impactor on the cord. Other AMs are compressive, applying an aneurysm clip to the cord, compressing the cord with forceps or inflating a balloon to achieve contusion-compression injury. The clinical relevance of transection injury AMs is doubtful, since most human SCIs are contusive-compressive. For further details, see recent reviews (16, 17).

## Surgical Treatment

### Humans

Mild SCIs may be managed conservatively. For severe SCI, conservative management was advocated in the early and mid-twentieth century (18), but the current trend is early surgery to eliminate the extradural cord compression and spinal instrumentation to stabilize the spine and prevent further cord damage. Whether early bony decompression improves neurological outcome is controversial (19–21). For central cord syndrome, some surgeons advocate surgical decompression, but others manage conservatively, without incontrovertible evidence favoring either approach (2).

### Dogs

There are two management options for dogs with acute SCI: conservative care with restriction of exercise for a few weeks or cross-sectional imaging (CT, MRI) and decompressive surgery (22). Conservative therapy is reserved for dogs with mild neurologic deficits or pain alone. Decompressive surgery aims to remove the extruded disc from the epidural space through “hemilaminectomy” (23), which has been the dominant surgical approach for the past 60–70 years, although more recent modifications have been described (24, 25). It is important to recognize progressive myelomalacia before it affects the phrenic nucleus in the cervical region, thereby causing suffocation, so that affected dogs can be euthanatized. Progressive myelomalacia is usually apparent within the first week after SCI and its development can be monitored by repeated examination of the panniculus (cutaneous trunci) muscle reflex (26).

## Evidence That the Dura Plays a Key Role

### Humans

**Figure 1:**
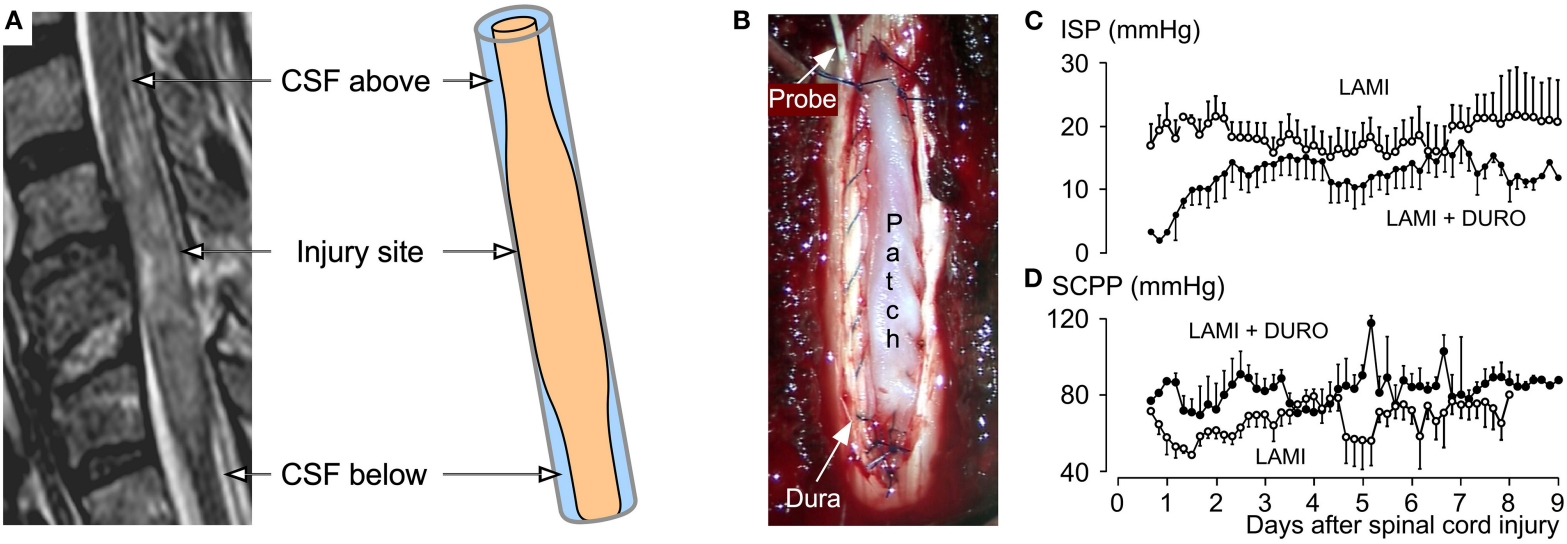
Intradural compartments after SCI and expansion duroplasty in humans. **(A)** (*left*) MRI and (*right*) Schematic: the three compartments are injury site (swollen cord compressed against dura), CSF above injury, CSF below the injury. **(B)** Sutured duroplasty patch and intradural pressure probe. **(C)** ISP (mean ± S.D.) and **(D)** SCPP (mean ± S.D.) vs. days after injury for 11 patients who had bony decompression plus stabilization (LAMI) and 10 patients who had bony decompression plus duroplasty plus stabilization (LAMI + DURO). Based on Phang et al. (33).

Spinal cord swelling is common after SCI with evidence that the amount of swelling, e.g., longitudinal cord signal change on T2 MRI, correlates with outcome (27). In a cohort of 65 SCI patients without bony compression, the extent of cord swelling against the dura increased with increasing severity of SCI and resolved slowly (t_1/2_ = 9 days) (28). At St. George's Hospital, we insert intradurally at the injury site a pressure probe to monitor intraspinal pressure (ISP) and spinal cord perfusion pressure (SCPP) at the injury site (29, 30). SCPP is mean arterial pressure minus ISP, analogous to cerebral perfusion pressure for brain injury. A key finding is that ISP remains high with low SCPP even after anterior and posterior bony decompression (29, 30) thus suggesting that the dura contributes to cord compression. ISP is generally high at the injury site with lower cerebrospinal fluid (CSF) pressure cranially and caudally, even after bony decompression (30–32). This finding suggests cord swelling against the dura at the injury site that results in three intradural compartments (Figure 1A): injury site (swollen cord compressed against dura), CSF compartment above and CSF compartment below the injury (34). Lumbar CSF pressure monitoring also supports the notion of cord compression against the dura at the injury site, because the lumbar CSF pressure signal is often non-pulsatile and because attempts to withdraw CSF result in a “dry tap” (32, 35, 36). An exploratory study (33) assessed safety and the effect on ISP and SCPP of duroplasty after SCI (Figure 1B) by comparing bony decompression in 11 patients vs. bony plus dural decompression in 10 (Figures 1C,D). Duroplasty was safe: 50% patients had no complications and 50% had non-compressive pseudo-meningocele that disappeared at 6 months, with no wound infection, no persistent cerebrospinal fluid (CSF) leak and no worsening neurological deficits. Compared with bony decompression alone, bony plus dural decompression significantly reduced ISP and increased SCPP. After bony decompression alone, 40% of the time, SCPP was very low (<60 mmHg likely to cause ischemic damage), compared with <5% of the time after bony plus dural decompression. A RCT is required to determine whether these improved physiological parameters at the injury site (lower ISP, higher SCPP) achieved by bony plus dural decompression improve functional outcomes. There are other reports of SCI patients treated with bony decompression plus duroplasty claiming improved outcome but without control patient groups (37, 38). SCI may be analogous to traumatic brain injury (TBI) where decompressive craniectomy requires not only bony but also dural decompression. The effectiveness of combined bony-dural decompression as a life-saving procedure after TBI has been shown in the RESCUEicp RCT (39). Overall, the SCI studies provide Level III evidence that duroplasty is beneficial.

### Dogs

**Figure 2:**
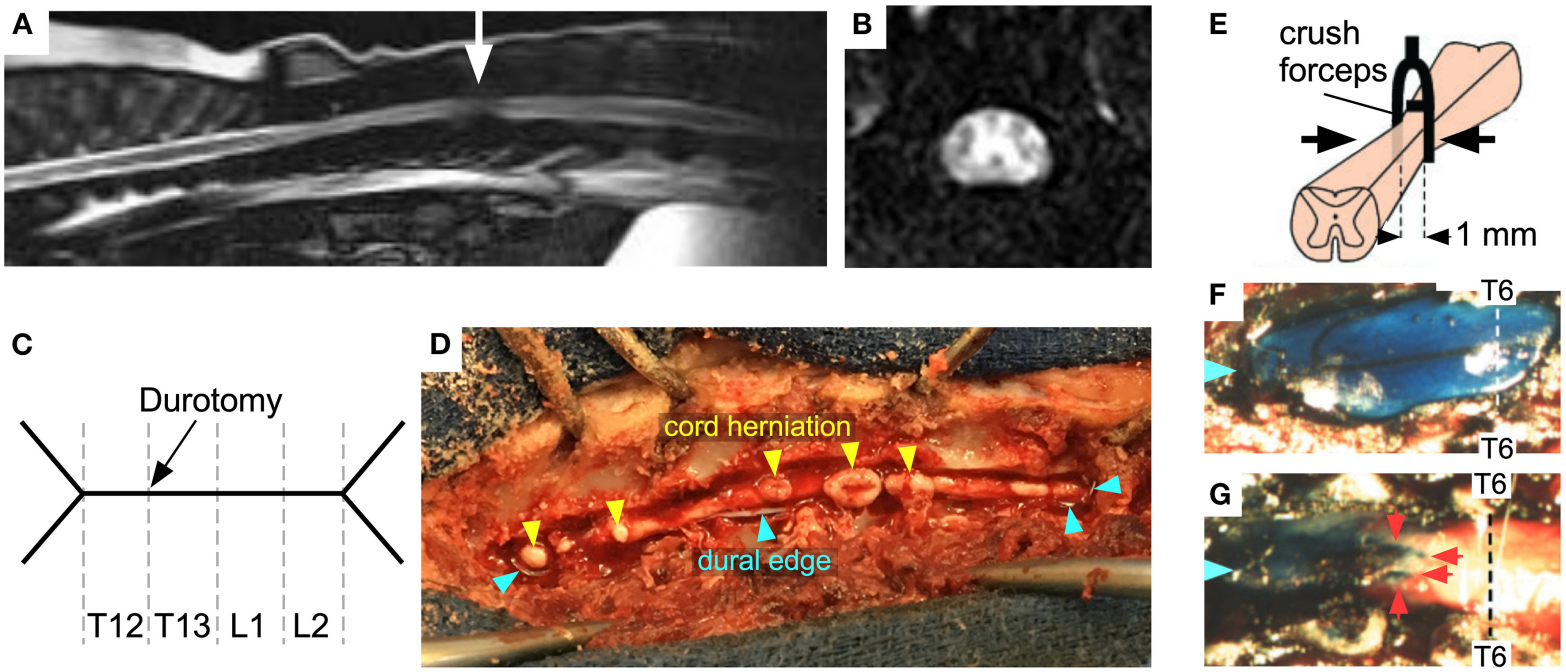
SCI in dogs and mice. **(A)** Sagittal, and **(B)** axial MRI of disc herniation at thoracolumbar junction (arrow) with cord swelling and cord compression against dura (loss of CSF signal around cord). **(C)** Schematic of durotomy in dog, Y-shaped at each end and extending over 4 spinal levels. **(D)** Dog cord herniation after dural opening. **(E)** Schematic showing forceps with spacer compressing mouse cord at T6. Exposed theca in **(F)** sham and **(G)** injured mice at 2 days after surgery: Evans blue dye was injected intracisternally (Blue arrows mark cranial end, red arrows indicate obstruction preventing the flow of dye). Adapted from (40).

Spinal cord swelling accompanies acute disc herniation in dogs, evident on myelography and MRI as loss of CSF signal (Figures 2A,B). Some studies suggest that the length of cord swelling on MRI (41) or extent of T2W hyperintensity within the cord (42) have prognostic value, though such analyses are confounded by lack of knowledge of the time between insult and scan. More recently, regions of T2W hypo-intensity have been shown to correlate well with failure to recover (13). The cord swelling after SCI raises the possibility that durotomy may reduce cord pressure thus improving cord perfusion (Figures 2C,D). Durotomy was initially used in experimental SCI in dogs and a benefit was suggested (43). Since then, durotomy has been reported sporadically by veterinarians (44, 45). Between 1950 and 1980, durotomy after acute SCI was primarily used as a prognostic tool for dogs in which there was acute complete sensorimotor loss (46). The dura was incised to evaluate cord consistency: animals with myelomalacia were considered irretrievable and thus euthanatized. In dogs the preferred procedure has hitherto been durotomy without duroplasty, because CSF leak has not been an issue.

In the past year, two reports on the use of durotomy for severe thoracolumbar SCI in dogs have been published (47, 48). Neither is a RCT, although the “baseline” rate of recovery for motor and sensory complete dogs is well-known (49–51). In one report, all dogs with motor and sensory complete SCI received a 4-vertebral length durotomy (48). This length was selected as a compromise between the typical length of loss of CSF signal on MRI/myelography and the need to minimize surgical time. At 6 months, 16 of the dogs recovered to walk, 6 did not recover (of which one developed progressive myelomalacia) and 4 were lost to follow-up. A comparison to expected recovery with Bayesian analysis suggested high likelihood of improved results over non-durotomy surgery.

The second study (47) included dogs with especially severe injuries: no pain perception in the hindlimbs plus extended region of T2W hyperintensity on preoperative MRI (42). Allocation into two groups was made according to the date of presentation. 25/65 (39%) dogs in the non-durotomy (control) surgery group recovered to walk vs. 29/51 (57%) dogs in the durotomy group. None of the dogs in the durotomy group developed progressive myelomalacia vs. 14/65 (22%) dogs in the control group. The length of durotomy was not specified.

### Other Mammals

In a mouse SCI model (40), which involves laminectomy and thoracic cord compression, the cord swelled against dura as evidenced by myelography (Figures 2E–G). ISP 48 h post-SCI was 30 vs. 10 mmHg in sham-operated mice. Since the mice had laminectomies, the high ISP must represent cord swelling against the dura. Reduced cord swelling in AQP4-null mice with SCI, evidenced by reduced ISP, was associated with improved neurological outcome assessed by footprint analysis, slope climbing and evoked potentials. There is now evidence from several SCI models in different species, including rat impact (52), rabbit balloon (53), and pig stretch (54) SCI models that, after the injury, the cord swells generating high ISP without bony compression. The degree of cord swelling depends on species, severity of injury, spinal level and mechanism of injury. Durotomy reduced ISP in stretched *ex vivo* pig cords from 35 to 10 mmHg (54). In a rat SCI impact model, duroplasty improved the walking score compared with no duroplasty and also reduced syrinx size (55). This finding suggests that duroplasty has additional benefit to reducing ISP; by reducing cord tethering to dura thus preventing syrinx formation. In a rat weight-drop SCI study, duroplasty led to white matter sparing compared with laminectomy (56). The above studies suggest that opening the dura after SCI is beneficial in multiple species and injury models.

A comparison of durotomy vs. duroplasty in rat contusion (55, 57) and rat compression (58) SCI models revealed reduced lesion volume with duroplasty because of less inflammation and less collagen scarring in the duroplasty group. In humans, duroplasty is preferred to durotomy because duroplasty reduces the risk of CSF leak through the wound but, in dogs, durotomy is more commonly performed. In a rat SCI study in which duroplasty initially appeared detrimental, it turned out that use of fibrin sealant swelled and caused cord compression (56). We thus advise against using fibrin sealants after duroplasty.

## Causes of Spinal Cord Swelling

In a balloon SCI model in rabbits, intraparenchymal hemorrhage developed within 5 hours and was the primary cause of cord swelling whereas edema became the primary cause of cord swelling at 5–7 days post-injury (59). Intraparenchymal hemorrhages and cord edema are also evident in human SCI and the length of cord edema and the length of intraparenchymal hemorrhage on MRI (27) as well the mean intraspinal pressure (60), monitored in the first week after SCI, correlate with outcome. Extravasation studies in dog models (61) and diffusion-weighted human MRI (62) suggest that cord edema after SCI is cytotoxic (cell swelling) and vasogenic (leaky capillaries) with accumulation of excess water intracellularly and interstitially. The water channel protein AQP4 plays a key role in water flow into and out of injured cord with opposing roles in vasogenic (63) vs. cytotoxic edema (40). Although histological studies in humans (64) and rodents (65) show large numbers of inflammatory cells at the injury site, it is unclear whether the volume of these cells contributes to cord swelling. There are, therefore, three causes of cord swelling after SCI: cord hematoma, cord edema and potentially, influx of inflammatory cells.

A key question is whether the swollen cord overcomes the pia to expand against dura. Spinal cords retrieved from fresh cadavers and immersed in water swelled generating intraparenchymal pressures, in the absence of dura, of 73.7 and 49.3 mmHg in the cervical and thoracic regions, respectively, that reduce to 9.7 and 10.3 mmHg post-piotomy (66). These findings suggest that the pia may contribute significantly to high intraparenchymal cord pressure after injury. However, the pia in these cords was intact. In humans with severe SCI, the cord appears expanded against the dura on MRI (28) and ISP is elevated (29, 30). In humans the pia is relatively tough; thus, the cord swelling evident on MRI after SCI likely indicates pial damage. This is supported by the observation that in a patient with severe SCI, simultaneously measured ISP and intraparenchymal pressures were elevated and equal (31). These findings suggest that in humans the intact pia is tough enough to limit cord expansion but, after severe SCI, the damaged pia no longer restricts cord swelling. In a compression SCI in rabbits, myelotomy (longitudinal incision of the cord to allow exit of hematoma and dead tissue) plus durotomy reduced ISP and improved histological outcomes compared with durotomy without myelotomy (67); however, the ISP was low (5–10 mmHg) in both animal groups without difference in hindlimb locomotor scores. In another rodent SCI model (68), durotomy alone or durotomy plus myelotomy improved several histological outcomes, but only durotomy alone promoted recovery of ladder walk performance and bladder function. In a rodent study of contusion SCI, the dura and pia contributed almost equally to increased ISP in the first 12 h but, by three days, the dura was responsible for the elevated ISP with little or no contribution from the pia (52). Based on these reports, the functional advantage of performing myelotomy, in addition to the durotomy, remain unclear with concern that myelotomy may itself produce cord damage.

## Evidence Against Cord Compression by the Dura

An MRI study of 184 motor-complete SCI patients concluded that adequate decompression is achieved in 91% patients with multi-level laminectomies alone (69). A patient's ISP varies widely (30, 32), with periods when lumbar CSF pressure is pulsatile and equals ISP (no cord compression against dura) and periods when lumbar CSF pressure is non-pulsatile and lower than ISP (cord compression against dura). Periods of non-pulsatile lumbar CSF after SCI have been observed by Kwon et al. (35). Thus, cord compression against dura is dynamic, i.e., a single MRI may not adequately assess such compression. In another study (70), laminectomy after SCI was not associated with low ISP. Aarabi et al. (69) proposed that cord compression against the dura may only occur in a small number or patients, provided multi-level laminectomies have been performed.

## Randomized Clinical Trials

Based on the above evidence, human and dog phase III studies are being set up to test durotomy as a potential surgical treatment for SCI.

### Humans

The trial, termed DISCUS (Duoroplasty for Injured cervical Spinal Cord with Uncontrolled Swelling), aims to recruit 222 adults with acute, cervical traumatic SCI who have surgery within 72 h. Eligible patients will have American spinal injuries association Impairment Scale (AIS) grade A, B, or C on admission. The patients will be randomized 1:1 to have spinal fixation plus laminectomy vs. spinal fixation plus laminectomy and duroplasty. The primary outcome will be change in AIS motor score at 6 months from baseline with multiple secondary outcomes assessing hand function, walking, urinary and anal sphincters as well as quality of life. DISCUS will have monitoring of ISP pressure and microdialysis from the injury site as optional extras. The study has been funded by the National Institute of Health Research (NIHR) and is currently being set up, aiming to start patient recruitment in July 2021 (https://fundingawards.nihr.ac.uk/award/NIHR130048). DISCUS is not yet registered.

### Dogs

A RCT in dogs is also being established. This will be a multinational multicentre study which aims to recruit 350 dogs with motor and sensory complete thoracolumbar spinal cord injuries. Dogs will be randomized 1:1 to receive either standard extradural decompression (removal of compressing herniated disc material) or undergo both extradural decompression and durotomy over 4 vertebral lengths. Outcome will be recorded as the proportion of dogs recovering to walk at least 10 steps without support (a routine measure of recovery of voluntary locomotion in dogs with severe thoracolumbar SCI). As a secondary outcome the proportion of dogs that develop progressive myelomalacia will also be compared between the two study arms.

## Discussion

Evidence from several animal species and from several types of SCI including contusion-compression in humans, disc prolapse is dogs as well as various experimental SCI models of contusion (weight drop, impactor) and/or compression (aneurysm clip, forceps, balloon) in other mammals suggest that the injured spinal cord swells against the dura. However, the human studies only provide Level III evidence. Some authors have suggested that multi-level laminectomies effectively decompress the cord without the need for duroplasty. Opening the dura after severe SCI improves cord physiological parameters in humans and animals and, in some rodent models, also improves histological and functional outcomes. Overall, we conclude that it is unclear whether opening the dura improves clinical outcomes in humans and dogs. The two RCTs being set up should provide Level I evidence.

## Author Contributions

SS wrote the sections on humans and part of the sections on rodents. NJ wrote the sections on dogs and part of the sections on rodents. Both authors have reviewed the entire manuscript.

## Conflict of Interest

The authors declare that the research was conducted in the absence of any commercial or financial relationships that could be construed as a potential conflict of interest.
